# The predictive value of SS-16 in clinically diagnosed Parkinson’s disease patients: comparison with ^99m^Tc-TRODAT-1 SPECT scans

**DOI:** 10.1186/s40035-016-0062-4

**Published:** 2016-08-20

**Authors:** Wenyan Kang, Fangyi Dong, Dunhui Li, Thomas J. Quinn, Shengdi Chen, Jun Liu

**Affiliations:** 1 Department of Neurology & Institute of Neurology, Ruijin Hospital affiliated to Shanghai Jiaotong University School of Medicine, Shanghai, China; 2 Department of Neurology, Ruijin Hospital North affiliated to Shanghai Jiaotong University School of Medicine, Shanghai, China; 3 Department of Radiation Oncology, Beaumont Health System, Royal Oak, MI 48073 USA

**Keywords:** Parkinson’s disease, DAT-SPECT, SS-16

## Abstract

**Background:**

Dopamine transporter based imaging has high diagnostic performance in distinguishing patients with Parkinson’s disease (PD) from patients with non-Parkinsonian syndromes. Our previous study indicated that the “Sniffin’ Sticks” odor identification test (SS-16) acts as a valid instrument for olfactory assessment in Chinese PD patients. The aim of the study was to compare the efficacy of the two methods in diagnosing PD.

**Methods:**

Fifty-two PD patients were involved in this study and underwent single photon emission computed tomography (SPECT) imaging using the labeled dopamine transporter radiotracer ^99m^Tc-TRODAT-1 to assess nigrostriatal dopaminergic function. Olfactory function was assessed with the “Sniffin’ Sticks” odor identification test (SS-16) in all patients who received DAT-SPECT scanning. Statistical analysis (SPSS version 21) was carried out to determine the diagnostic accuracy of SS-16 as well as its correlation with ^99m^Tc-TRODAT-1 SPECT, its positive predictive value (PPV), and negative predictive value (NPV).

**Results:**

We identified a negative correlation between SS-16 and DAT SPECT (Kappa = 0.269, *p* = 0.004). By using the ^99m^Tc-TRODAT-1 uptake results as the gold standard, the sensitivity and specificity of SS-16 was 56.8 and 37.5 %, respectively. Furthermore, the negative and positive predictive values were calculated as 13.6 and 83.3 %, respectively.

**Conclusions:**

SS-16 would not be used as a diagnostic tool for early stage PD patients. Negative results of SS-16 would not exclude the diagnosis of PD. Further tests are needed for validation.

## Background

At least 50 % of nigrostriatal neurons have already been lost at the time of clinical diagnosis for Parkinson’s disease (PD), a diagnosis which significantly relies on the identification of classical motor symptoms. An early and precise diagnosis of PD is critical since early treatment with neuroprotective agents before most substantial neuronal loss has occurred is believed to be beneficial which could potentially slow or prevent the development of PD symptoms.

As one of the most common non-motor symptoms, a majority of PD patients are diagnosed with olfactory impairment, which may even appear before the development of motor symptoms. Therefore, analysis of olfaction may be a useful diagnostic tool for PD. There are two main olfactory tests available that can readily be utilized in PD patients, the University of Pennsylvania 40-item smell identification test (UPSIT-40) and the “Sniffin’ Sticks” 16-item identification test SS-16 [1]. Tests of odor identification might help differentiate PD patients from healthy controls and have many advantages, including: low cost, ease of administration and rapid results. Therefore, olfactory tests are suited for routine use in daily clinical practice and are currently utilized in Europe. Our previous study indicated that SS-16 act as a valid instrument for olfactory assessment in Chinese PD patients, and hyposmia may correlate with autonomic dysfunction in patients with PD [2].

In recent years, studies have demonstrated that a steady degradation of striatal uptake was observed as a function of clinical duration after an estimated preclinical duration of eleven years prior to PD symptom onset [3]. Several different diagnostic tools have been evaluated for their ability to detect nigrostriatal cell loss. The most widely used tests are dopamine transporter single-photon emission computed tomography (DAT SPECT), [18 F]DOPA positron emission tomography (PET), and transcranial sonography (TCS) [4]. A recent systematic review indicated that sensitivity and specificity of DAT SPECT imaging to detect nigrostriatal cell loss were 98 %, and it seems to be accurate in detecting nigrostriatal cell loss in patients with Parkinsonism [4]. SPECT-based brain scans have become increasingly common in routine PD diagnosis [2, 5, 6]. However, these scans are expensive and there are still technical issues which can lead to difficulties with interpretation in borderline cases. In this study, using DAT SPECT results as the clinical gold standard, we assessed the diagnostic accuracy of SS-16 on 52 patients diagnosed with PD based on clinical features in their early stage. Furthermore, this enabled us to also assess the ability of hyposmia defined by a SS-16 score to predict a DAT deficit.

## Methods

### Participants

A total of 52 PD patients were recruited in this study by consecutive referral at the movement disorders clinic in the Department of Neurology, Ruijin Hospital affiliated to Shanghai Jiao Tong University School of Medicine. Two senior movement disorders specialists clinically identified all patients in this study according to the United Kingdom Parkinson’s Disease Society Brain Bank criteria. Patients with possible olfactory dysfunction secondary to other causes, such as nose surgery, chronic sinusitis, acute upper respiratory tract infection and other central nervous system diseases were excluded. Moreover, illiterate individuals were excluded from this study. All patients underwent the Chinese equivalent of the Mini Mental State Examination. We excluded participants with scores less than 20 for individuals that received 6 years or less of primary school education and participants with scores less than 24 for individuals that received more than 6 years of schooling. All subjects read and signed an informed consent prior to participation in the study. This study was approved by the Ethics Committee of Ruijin Hospital affiliated to Shanghai Jiao Tong University School of Medicine.

### Experimental procedure

#### ^99m^Tc-TRODAT-1 SPECT

^99m^Tc-TRODAT-1 SPECT was performed as described in our previous study [7]. Briefly,two hours after intravenous injection of ^99m^Tc-TRODAT-1, prepared from a pre-formulated kit provided by Jiangsu Nuclear Medicine Institute (WuXi city, China), ^99m^Tc-TRODAT-1 uptake was assessed by SPECT imaging. Images were acquired via a double-headed gamma camera (Simens, Symbia T16), with a 140 ± 14 keV energy window. Acquisition time for the projection was 30s, with a 1.45 zoom and 3 mm slice thickness at the level of the basal ganglia. Regions of interest (ROIs) for ^99m^Tc-TRODAT-1 uptake were established by MRI (1.5 T, Siemens, Germany). Of note, if the average ^99m^Tc-TRODAT-1 uptake was less than 1.21 for the right side or 1.31 for the left side, striatal uptake (a combination of putamen and caudate uptake) of the alternative side was considered pathological [8].

#### Odor identification test

Bilingual university graduate students translated the original SS-16 to Chinese (simplified) [9]. The content and wording of the questionnaires were discussed with the patients’ representatives and caregivers, as well as public health experts. and the 16 odors in the original “Sniffin’ Stick” odor identification test were kept the same but alternative descriptions were developed to accommodate the Chinese population. For example, the descriptions “pine tree” and “grapefruit” were replaced with the Mandarin equivalent of “wood,” and “pomelo”, which are common in mainland China and are similar odors. The test was continuously revised until it was considered unambiguous by the research committee. The odor sticks were placed approximately 2 cm in front of the nose of the subjects to smell for 3 s, and the subject was required to choose between four alternatives in a forced-choice paradigm. The interval between tests of various odors was about 30s. Each correct answer for each odor received a score of 1; thus, the total score expressed as the sum of correct responses ranged from 0 to 16. Subjects with scores lower than 9.5 were defined as olfactory impaired [2].

### Statistical analysis

Statistical analysis was carried out using SPSS version 21. The diagnostic accuracy of SS-16 was determined by comparing their results to the surrogate gold standard: ^99m^Tc-TRODAT-1 scans. Diagnostic accuracy is defined as the sensitivity, specificity, positive predictive value (PPV) and negative predictive value (NPV). Finally, we used Kappa test to compare the consistency of the two examinations. P values of less than 0.05 were considered statistically significant.

## Results

### Descriptives

The mean age of the 52 patients was 60.65 ± 7.82 years, and the majority (32/52) were male. The mean course of disease was 4.04 ± 3.54 years, and the mean Hoehn and Yahr score was 1.63 ± 0.83. The average UPDRS III score was 14.18 ± 11.23. The R/L-Striatal 99mTc-TRODAT-1 uptake was 0.81 ± 0.68. All recruited PD patients had the 16-item odor identification test from Sniffin’ Sticks (SS-16), the score was 8.60 ± 3.16.

### Accuracy of SS-16 in clinically diagnosed PD patients

**Fig. 1:**
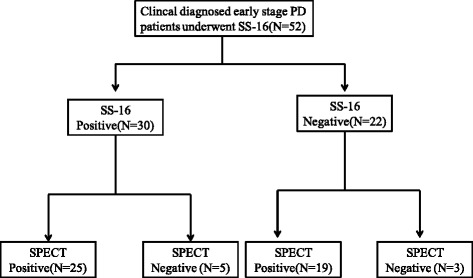
The flowchart of categorization of subjects

For the complete overview of the results of the SS-16, ^99m^Tc-TRODAT-1 SPECT in each subgroup of patients, see Fig. 1.

In the 52 subjects (20 women, 32 men) who underwent the 16-item odor identification test from Sniffin’ Sticks (SS-16), 30 subjects (57.69 %) had distinct hyposmia (SS-16 scores <9.5). Of the 30 patients, 25 (83.33 %) had an abnormal 99mTc-TROD AT-1 uptake scan. 22 of the 52 patients had normal olfactory dysfunction, while only 3 had a normal ^99m^Tc-TRODAT-1 uptake scan. Taking ^99m^Tc-TROD AT-1 uptake results as the gold standard, the sensitivity of SS-16 was 56.8 % (25/(25 + 19)), and the specificity was 37.5 % (3/(5 + 3)).

### Predictive value of SS-16 for the results of the DAT SPECT scans

To estimate the predictive values of SS-16 for the early-diagnosis of PD, all 52 patients were included in the analysis. In 28 patients (53.85 %), the result of the SS-16 was in accordance with the result of the ^99m^Tc-TROD AT-1 scan. The PPV of a positive SS-16 test result for an abnormal scan was 83.3 % (25/30). However, the NPV of a negative SS-16 test result for a normal scan was only 13.6 %(3/22). The false negative rate of SS-16 test result for the DAT imaging result was 43.2 % (19/44). SS-16 and DAT-SPECT were both positive in 25 patients (48.1 %), while 3 patients (5.8 %) had double negative results. In summary, in 28 patients, the results of the SS-16 test were in accordance with the results of the DAT SPECT scan (Kappa test, Kappa = 0.269, *P* =0.004). Therefore, the consistency was poor (see Table 1).

**Table 1:** Predictive values of SS-16 for the results of the DAT-SPECT scan

	Sensitivity (%)	Specificity (%)	Positive predictive value (%)	Negative predictive value (%)	Kappa	*P* value
SS-16	56.8 %	37.5 %	83.3 %	13.6 %	0.269	0.004

## Discussions

Olfactory dysfunction is a prodromal symptom in PD and can be found several years before the appearance of motor signs. In addition, it develops independent of treatment or age at onset. Olfactory tests, including ethnically specific odors, were recommended by the European Federation of Neurological Societies and the Movement Disorder Society as screening tests for pre-motor PD [10]. Moreover, the fact that α-synuclein deposits are present in the olfactory bulb and anterior olfactory nucleus at Braak stage I explains its high sensitivity in early PD [11]. A study that correlated diffusion tensor imaging (DTI) with olfactory deficit in early PD patients demonstrated that the Fractional anisotropy (FA) values of White Matter (WM) were significantly reduced in the early PD group versus healthy controls [12].

Prior studies have demonstrated SS-16 reliability in differentiating PD patients and healthy subjects in Asia and Latin America [1, 2]. Furthermore, a previous study suggested that the performance of Sniffin’ Sticks was better than that of UPSIT, even among Children to whom discriminating different smells is more perplexing [7]. Thus, SS-16 showed capability in differentiating olfactory impairment in spite of the poor life experience of different smells. In the present study we try to investigate whether Sniffin’ Sticks hold the qualification to detect early PD clinically with DAT SPECT as a gold standard.

Previous studies focus on identifying the diagnostic value of prodromal or early PD by combining smell tests with imaging examinations [13, 14]. Nevertheless, the investigation of consistency between olfactory impairment results and imaging diagnosis standards is lacking. In the present study, we found that the correlation between these two items is negative (Kappa = 0.269, *p* = 0.004). Taking ^99m^Tc-TROD AT-1 uptake results as the gold standard, the sensitivity of SS-16 for DAT SPECT was 56.82 % with a false negative predictive value of 43.2 %. The negative predictive value was 13.6 %, and the specificity was only 37.5 %, while the positive predictive value was 83.3 %. The over misdiagnosis was reasonable to some degree and a number of factors might contribute to this outcome.

First, although olfactory impairment acts as a symptom of PD originating from extranigral neuropathological change according to Braak staging of Parkinson’s disease, hyposmia is prevalent in Parkinson’s disease with the deficiency of smell test ranging from 73 to 90 %, indicating a minority of PD patients not suffering from olfactory deficiency [15]. In a recent study, the utilization of SS-16 was compared among different countries with China included. The results suggested that the scores of several items called “component1” were significantly different across PD patients from different country groups because of culture, smell familiarity and environmental variations [11, 16]. Our previous study used the adapted SS-16 translational version evaluation (mentioned in the methods) in Chinese patients, resulting in a sensitivity of 86 % and the specificity of 81 % in discriminating PD patients from healthy controls. Among the 110 patients, 66.4 % were identified as having hyposmia. In addition, the cohort of 52 patients was in the early stage (Hoehn and Yahr score was 1.63 ± 0.83), thus, 57.7 % could be explained in the study and may not affect the results [2]. Thus, a certain rate of false negative and positive value exists in the particular SS-16 test and among a certain number of Chinese population. To that end, a larger sample size to evaluate the SS-16 test is in urgently needed to demonstrate stable scale characteristics.

Second, in the present study, the clinical PD diagnosis fulfilled the established UK Parkinson’s Disease Society Brain Bank clinical diagnostic criteria. The clinical symptoms of PD were assessed by movement disorder specialists. Nevertheless, some atypical parkinsonism related disorders, such as MSA, essential tremor progressive supranuclear palsy and cortico-basal degeneration, might not be clearly discriminated from PD in the early disease course. However, olfactory function among the above diseases is impaired mildly and for monogenic PD, especially in recessive forms, the dysfunction is less serious than in PD. Consequently, there is a tendency for false negative predictive value elevation and with reduced specificity.

Third, with regard to DAT-SPECT diagnosistic capability, M. Menendez-Gonzalez [17] concluded that false negative values would become higher due to the quantitative analysis of SPECT scans and the existence of SWEDDs (scans without evidence of dopaminergic deficits). In addition, in early PD, 15 % patients might show normal brain scan results. The misdiagnosis could not be excluded in the assessment.

The present study is the first to take DAT SPECT as the gold standard to assess the correlation between SS-16 scores and ^99m^Tc-TROD AT-1 uptake results from SPECT imaging in a Chinese population. The positive predictive value against SPECT was 83.3 %. Our previous study compared TCS with SPECT, finding the positive predictive value to be 91.7 % which was not much higher than SS-16 [8]. However, negative results of the SS-16 test could not exclude the diagnosis of PD. And there are some limitations in our study. For one thing, healthy subjects were not enrolled in the study. In addition, this study is limited by the small sample size of 52 patients, and larger samples are necessary in the future to produce results more representative of the population.

## Conclusions

In conclusion, SS-16 is a non-invasive, low cost, rapid and convenient procedure recommended to be used as a clinical test in spite of the negative correlation with dopamine uptake results reflected via DAT SPECT. SS-16 would not be used as a diagnostic tool for early stage PD patients as a negative SS-16 test result could not exclude the diagnosis of PD.
